# Knowledge, attitude, and practice related to COVID-19: A comparison between patients with mental illness and the general population in Qatar

**DOI:** 10.3389/fpsyt.2022.1013096

**Published:** 2022-10-20

**Authors:** Suhaila Ghuloum, Ibrahim Makki, Yassin Hassan Eltorki, Oraib Abdallah, Fahad Farhan Alanzy, Mohamed Adil S. Khoodoruth, Mohamed F. Ali, Hassen Al-Amin

**Affiliations:** ^1^Department of Psychiatry, Hamad Medical Corporation, Doha, Qatar; ^2^Department of Psychiatry, Weill Cornell Medicine, Doha, Qatar

**Keywords:** KAP, COVID-19, mental health, patients, public

## Abstract

**Background:**

In 2020, the World Health Organization (WHO) declared COVID-19 a global health pandemic. The rapid spread and high fatalities associated with COVID-19 have increased interest in assessing Knowledge, Attitude, and Practice (KAP) toward this illness among the general population in comparison to specific subgroups. Most publications to date have explored KAP among the general public, healthcare providers, and people with chronic conditions, but not amongst those with mental illness. Yet, research has shown patients with mental illness are at higher risk of poor outcomes related to infectious diseases such as COVID-19. The objective of this study is to compare KAP toward COVID-19 between people with mental illness and the general public.

**Materials and methods:**

This is a cross-sectional study, done over 3^°^months in 2020, to compare KAP during the COVID-19 pandemic in three groups: outpatients from outpatient Psychiatry clinics (*N* = 165), inpatients admitted to a Psychiatry ward (*N* = 100), and the general public (*N* = 345). KAP parameters were assessed through online surveys.

**Results:**

The proportion of subjects in the public group (84.8%) giving the correct responses to most Knowledge questions was significantly higher than those in the inpatient and outpatient groups. Compared to the public and inpatient groups, subjects in the outpatient group (92.7%) were significantly more optimistic and confident that COVID-19 would be brought under control. A higher proportion of subjects from the general public (82.9%) indicated that they attended crowded places and were more compliant in wearing masks. Multiple linear regression analyses showed that poorer COVID-19 knowledge was associated with being single and having a young age (18–29), with both inpatients and outpatients and with primary-or secondary-level education.

**Conclusion:**

Patient populations, both inpatients and outpatients, had inadequate Knowledge, more positive attitudes and confidence regarding the outcome of COVID-19, and less safe practices than the public. This highlights the need for targeted approaches around COVID-19 and pandemics in general in this vulnerable population.

## Introduction

On March 11, 2020, the World Health Organization (WHO) declared a global health emergency due to the COVID-19 pandemic. Although COVID-19 is considered to be less fatal than other virally-transmitted diseases, such as the Severe Acute Respiratory Syndrome (SARS) and Middle East Respiratory Syndrome (MERS), it is highly infectious, which explains its rapid spread internationally. As of August 2, 2022, the WHO has reported a total of 575,887,049 confirmed cases of COVID-19 infections globally, including 6,398,412 deaths (1).

The rapid spread and the high number of fatalities caused by the disease have prompted most countries to implement several precautionary measures to contain it. Such measures ranged from practicing hand and respiratory hygiene and social distancing to total national lockdowns. The success of such governmental efforts and whether people adhere to them and adopt the required behavioral changes depends largely on the public’s Knowledge, Attitude, and Practice (KAP) toward COVID-19 (2, 3). This has given rise to increased interest in assessing KAP among the general population in comparison to specific subgroups, with varying outcomes of such assessments from one population to another. For instance, at the early stages of the pandemic, Zhong et al. (2) explored KAP among the population of Wuhan. They concluded that their sample demonstrated a high rate of Knowledge about the virus, an optimistic Attitude, and a high level of adherence to safety Practices (2). The authors, recognizing the association of higher Knowledge with more positive Attitudes and preventive methods, acknowledge that these results may not be generalized to a population of different demographics. On the other hand, other research assessing KAP concluded that their samples had lower levels of Knowledge and poor practices regarding COVID-19, urging local authorities to establish awareness programs to improve KAP among the general population (4–6).

Research has noted the urgency of identifying vulnerable populations, highlighting the importance of targeting these groups with awareness and preventive efforts. People with mental illness (PWMI) are more vulnerable during pandemics and have a higher risk of becoming infected (7). Causes may include cognitive impairment, lack of awareness of risks associated with infections and of protective measures, and a lower ability to adhere to protective measures due to the nature of their illness or living circumstances (8). People with severe mental illness often have lower educational attainment and health literacy and may have lesser social support (9). Those who are admitted to hospital or residential care are often in shared spaces that may be overcrowded. Recent data has indicated that people with mental illness who contract the COVID-19 virus have poorer outcomes (10). Smoking, poor sleep quality, psychotropic medication side effects and the sedentary lifestyle often associated with PWMI result in a higher prevalence of obesity, cardiovascular disease and chronic obstructive pulmonar disease; all contributing factors to the higher risk of COVID-19 (11, 12). Despite these trends, most publications to date have explored KAP among the general public, healthcare providers, and vulnerable groups such as people with chronic conditions, but not in the mental health population (13–18). We found one paper that used the Knowledge, Attitude and Practices questionnaire on a convenient sample of 200 patients with mental illness attending a psychiatry hospital. Their results show 51.5% of their participants had poor knowledge, 75% moderate attitude, and 61% low to moderate practices toward the pandemic (19). This group is considered highly vulnerable due to many factors that might impede their adherence to preventive measures for COVID-19, putting them at higher risk of infection and poorer outcomes (9).

Qatar, like other countries, has been impacted by COVID-19, with a total of 3,758,024 confirmed cases and 681 deaths as of August 2, 2022 (20). The government has taken many steps to ensure that knowledge is disseminated to all residents of Qatar in different languages, targeting the large multi-ethnic population. Health agencies have dedicated their social media accounts to sharing information about the virus with the public and providing better education on safety measures. The objective of this study was to explore the efficacy of these efforts in raising public KAP toward COVID-19 and to compare it to the KAP among people with mental illness, with the expectation that PWMI would show significant deficits in their knowledge of the COVID-19 pandemic and its prevention compared to the general population. Despite their established vulnerability, barely any publication exploring KAP of the COVID-19 pandemic in PWMI was identified.

## Materials and methods

### Study design, setting, and participants

This is a cross-sectional study comparing the Knowledge, attitude, and safe practice (KAP) during the COVID-19 pandemic in three groups: Psychiatry outpatients, Psychiatric ward inpatients, and the general population. Using online surveys, the study was conducted between May and August 2020 with the approval of the Research Office and Ethical Committee of Hamad Medical Corporation (HMC) (Reference number: MRC-05-050). Participants signed an online or manual informed consent form. Psychiatry patients were recruited from two facilities: Mental Health Hospital (MHH) in Doha, Qatar, and Al-Khor Hospital (AKH) in Al-Khor, Qatar. The general population was invited through messages sent by the telecommunications company Ooredoo which contained a link to an online survey.

The inclusion criteria for patients were (i) having a mental illness, (ii) attending the outpatient clinics or being admitted to the inpatient services during the study period, (iii) ages 18–65 years inclusive, and (iv) having the capacity to sign the consent form. The exclusion criteria included having the diagnosis of a learning disability and patients admitted from prison. The inclusion and exclusion criteria for the general population were (i) adults aged 18–65 and (iii) Arabic or English speaking.

Sample size was estimated based on the proportion of correct answers for each of the three groups using Chi-Square tests, utilizing three pairwise comparisons. Limits were set to a difference of 33% for each comparison, with a confidence interval of 90%, a power of 80%, and a type one error of 5% (significance level). A minimal sample size of 85 subjects in each group was needed to reach significance. Factoring a potential 20% dropout rate, 100 subjects were targeted for each subgroup.

### Study procedure and measures

The inpatient group was recruited from the inpatient units of the Mental Health Services in Doha. These units are the only available inpatient psychiatric facility in Doha. The researchers invited all inpatients who fulfilled the eligibility criteria. Participants signed an informed consent form and answered a questionnaire. Outpatients attending the service within the same period (May-August 2020) who met the criteria were randomized by the lead PI and distributed among the team members. In this subgroup, consent was obtained by phone, the standard method of contacting patients during the pandemic. The research members read a script (approved by the ethical committee) explaining the research on a phone calls and then obtained verbal consent to participate. For the general population, the telecommunications company randomly sent a text message to Arabic and English speakers in their records, linked to an online questionnaire (available on SurveyMonkey, San Mateo, CA, USA), inviting them to participate in the study.

Participants answered both a sociodemographic questionnaire and the KAP instrument. The latter was published in English based on a sample from the general public in Wuhan, China (2).

The KAP instrument was translated for an Arab-speaking population, utilizing the back-translation method, whereby three bilingual (English and Arabic) team members independently translated the instrument from English to Arabic. A final Arabic version was agreed upon by all three translators. This version was piloted in ten patients and staff to ensure that the items were clear and understandable. The three translators reviewed the input from the pilot participants and agreed on the proper modifications accordingly. The Arabic version was back-translated to English and independently reviewed by another team member to ensure the contents were consistent with the original instrument and with the Ministry of Public Health information in Qatar on the COVID-19 pandemic and its prevention.

The English and Arabic versions of the KAP instrument include 16 items divided into three sections: (1) Twelve items covering Knowledge about COVID-19, where participants can answer each question with “True,” “False,” or “I do not know.” (2) Two questions related to Attitude: the first question asking whether the respondent agrees that the pandemic will be controlled with the optional answers: “Yes,” “No,” and “I do not know,” and the second question asking whether they trust Qatar to control the spread of COVID-19, with binary answers Yes vs. No. (3) Two items covering the subjects’ practice during the pandemic: going to crowded places (Yes vs. No) and wearing a mask when going out (Yes vs. No).

### Data analysis

Data were analyzed using IBM Statistical Package for Social Sciences (IBM SPSS^®^ Version 24; IBM Corp, USA). The significance level was set at 0.05. The categorical data on sociodemographics and the correct answers on each instrument’s item are presented as percentages. The Chi-Square test compared the proportions between the three groups of participants with Bonferroni corrections for multiple comparisons.

The total score on COVID-19 Knowledge was calculated from the sum of correct responses on the first 12 questions. A correct answer received a score of 1, an incorrect answer or “I do not know” received a 0. The total knowledge score (0–12) (continuous variable) between the three groups was analyzed with the Analysis of Variance test (ANOVA). Each of the two questions on Attitude was dichotomized (Yes vs. No) to the questions.

Multiple linear regression analysis checked if the differences in the total Knowledge score (dependent variable) between the three groups were still valid even after controlling for all the sociodemographic factors (the independent variables entered as dummy variables in SPSS) listed in Table 1. Multivariable logistic regression to assessed whether the variations in the three groups’ COVID-19 attitudes and practices were still valid after controlling for all the independent sociodemographic factors. Four analyses were conducted, utilizing the backward method for each of the questions on the attitudes and practices as the dependent variable and the sociodemographic categorical factors as the independent variables. A Nagelkerke Pseudo R2 test to was added to assess the goodness of fit of the regression models.

**TABLE 1:** Sociodemographic characteristics of the sample by group.

	Public *N* = 345	Outpatient *N* = 165	Inpatient *N* = 100
**Gender**			
Female	176 (51.0%)^c^	72 (43.6%)^c^	18 (18.0%)
Male	169 (49.0%)	93 (56.4%)	82 (82.0%)^a,b^
**Age-group (years)**			
18–29	53 (15.4%)	41 (24.8%)^a^	40 (40.0%)^a,b^
30–49	205 (59.4%)	84 (50.9%)	50 (50.0%)
50–65+	87 (25.2%)^c^	40 (24.2%)^c^	10 (10.0%)
**Marital status**			
Married	246 (71.3%)^c^	112 (67.9%)^c^	40 (40.0%)
Others	15 (4.3%)	4 (2.4%)	12 (12.0%)^a,b^
Single	84 (24.3%)	49 (29.7%)	48b (48.0%)^a,b^
**Education**			
Primary school	7 (2.0%)	15 (9.1%)^a^	25 (25.0%)^a,b^
Secondary school	54 (15.7%)	55 (33.3%)^a^	35 (35.0%)^a^
College degree	201 (58.3%)^b,c^	77 (46.7%)^c^	30 (30.0%)
Masters or PhD	83 (24.1%)^b,c^	18 (10.9%)	10 (10.0%)
**Occupation**			
Employed	251 (72.8%)^b^	93 (56.4%)	61 (61.0%)
Retired	9 (2.6%)	10 (6.1%)	8 (8.0%)^a^
Student	24 (7.0%)	8 (4.8%)	1 (1.0%)
Unemployed	61 (17.7%)	54 (32.7%)^a^	30 (30.0%)^a^
**Place of current residence**			
Doha	285 (82.6%)^c^	129 (78.2%)	7 (70.0%)
Other parts of Qatar	60 (17.4%)	36 (21.8%)	30 (30.0%)^a^

^a^Significantly higher than the Public group, ^b^significantly higher than the Outpatient group, ^c^significantly higher than the Inpatient group.

Tests were adjusted for all pairwise comparisons within a row using the Bonferroni correction.

## Results

The total number of eligible inpatients approached was 120, of whom 100 participated. Of 211 eligible outpatients, 165 finished the survey. Ooredoo, a Qatari-based telecommunications company sent 10,000 phone messages to the general population, of whom 345 completed the survey.

### Sociodemographic characteristics of the sample by group

The majority in the sample were males (56.4%), aged 30–49 years (55.6%), were married (65.2%), had a university or college degree (68.7%), were employed (66.4%), and resided in Doha (79.3%). There were significant sociodemographic differences between the three groups: Gender (χ^2^ = 34.36, df = 2, *p* < 0.001), Age-group: (χ^2^ = 32.72, df = 4, *p* < 0.001), Marital status (χ^2^ = 38.58, df = 4, *p* < 0.001), Education (χ^2^ = 102.96, df = 6, *p* < 0.001), Occupation (χ^2^ = 29.30, df = 6, *p* < 0.001), and Place of residence (χ^2^ = 7.71, df = 2, *p* = 0.02). *Post hoc* comparisons (Table 1) showed that the proportion of males in the inpatient group was significantly higher than those in the other two groups. The percentage of young subjects (aged 18–29) was significantly higher in the patients’ groups than in the public group. The proportion of subjects with university or college degrees was significantly higher in the public group than the two patients’ groups. The latter showed a significantly higher percentage of unemployed subjects than the ones participating from the general public.

### Knowledge, attitude, and practice survey responses by group

Of the 12 questions on Knowledge, only two of the answers (on questions 7 and 8) showed no significant differences in Knowledge (spread through respiratory droplets and prevention by wearing masks) among the three groups. In general, the number of subjects in the public group giving the correct responses to all the other ten Knowledge questions was significantly higher than those in the inpatient group (Table 2). The percentage of subjects with correct answers to Knowledge questions 2, 6, and 9 were significantly higher in public than in the outpatient group only. The percentage of subjects giving correct answers to questions 1, 5, 10, and 12 in the outpatient group was significantly higher than in the inpatient group only. The proportion of subjects in the outpatient group giving the correct answers to questions 3, 4, and 11 was not statistically significant from the public and inpatient groups (Table 2). ANOVA showed that the total knowledge score differed significantly between the three groups, *F*(2, 609) = 41.57, *p* < 0.001. *Post hoc* comparisons showed the score was statistically higher in the public group than the other two groups (*p* < 0.001), and the one in the outpatient group was higher than the one for the inpatient group (*p* < 0.001) (Table 2).

**TABLE 2:** COVID-19 knowledge, attitude, and practice by group.

	Public *N* = 345	Outpatients *N* = 165	Inpatients *N* = 100
	
Knowledge	Correct answers n (%)		
1. The main clinical symptoms of COVID-19 are fever, fatigue, dry cough, and myalgia.	317 (91.9%)^c^	150 (90.9%)^c^	76 (76.0%)
2. Unlike the common cold, stuffy nose, runny nose, and sneezing are less common in persons infected with the COVID-19 virus.	219 (63.5%)^b,c^	75 (45.5%)	47 (47.0%)
3. Currently, there is no effective cure for COVID-19, but early symptomatic and supportive treatment can help most patients recover from the infection.	323 (93.6%)^c^	150 (90.9%)	81 (81.0%)
4. Not all persons with COVID-19 will develop severe cases. Only those who are elderly, obese, and have chronic illnesses are more likely to be in severe cases.	251 (72.8%)^c^	112 (67.9%)	57 (57.0%)
5. Eating or contacting wild animals would result in infection by the COVID-19 virus.	213 (61.7%)^c^	91 (55.2%)^c^	31 (31.0%)
6. Persons with COVID-19 cannot infect others with the virus when a fever is not present.	283 (82.0%)^b,c^	100 (60.6%)	51 (51.0%)
7. The COVID-19 virus spreads *via* respiratory droplets of infected individuals.	314 (91.0%)	139 (84.2%)	85 (85.0%)
8. Ordinary residents can wear general medical masks to prevent infection by the COVID-19 virus.	295 (85.5%)	146 (88.5%)	94 (94.0%)
9. Children and young adults do not need to take measures to prevent the infection by the COVID-19 virus.	327 (94.8%)^b,c^	141 (85.5%)	74 (74.0%)
10. To prevent the infection by COVID-19, individuals should avoid going to crowded places such as train stations and avoid taking public transportations.	334 (96.8%)	163a (98.8%)^c^	93 (93.0%)
11. Isolation and treatment of people who are infected with the COVID-19 virus are effective ways to reduce the spread of the virus.	338 (98.0%)^c^	159 (96.4%)	93 (93.0%)
12. People who have contact with someone infected with the COVID-19 virus should be immediately isolated in a proper place. In general, the observation period is 14 days.	337 (97.7%)^c^	157 (95.2%)^c^	82 (82.0%)
Knowledge Total score (mean ± standard deviation)	10.29 ± 1.49^b,c^	9.59 ± 1.65^c^	8.6 ± 2.07
**Attitude**	Positive attitude		
13. Do you agree that COVID-19 will finally be successfully controlled? Yes	247 (71.6%)	140 (84.8%)^a,c^	70 (70.0%)
14. Do you have confidence that Qatar can win the battle against the COVID-19 virus? Yes	275 (79.7%)	153 (92.7%)^a^	88 (88.0%)
**Practice**	Correct practice		
15. In recent days, have you gone to any crowded place? No	286 (82.9%)^b,c^	118 (71.5%)	69 (69.0%)
16. In recent days, have you worn a mask when leaving home? Yes	338 (98.0%)^c^	158 (95.8%)^c^	84 (84.0%)

^a^Significantly higher than the Public group, ^b^significantly higher than the Outpatient group, ^c^significantly higher than the Inpatient group.

Tests were adjusted for all pairwise comparisons within a row using the Bonferroni correction.

The outpatient group was significantly more optimistic about possible control of COVID-19 (item 13) than the public and inpatient groups. Likewise, outpatients responded with more confidence than the other two groups in Qatar, agreeing that Qatar would be able to win against COVID-19 (item 14) (Table 2). The majority of the public group participants indicated that they had not attended crowded places in recent days (article 15). This percentage was significantly higher than those of the two patients’ groups. The subjects in the public and outpatient groups were significantly more compliant in wearing masks when leaving the house than those in the inpatient group (item 16) (Table 2).

### Predictors of the total score on COVID-19 knowledge

The multiple linear regression analysis (Table 3) showed that both the inpatient and outpatient groups were associated with lower total Knowledge in Qatar even after controlling for the various sociodemographic factors. The model tested was a good fit as the R2 was 0.18, and the F change (9.56) was significant (*p* < 0.001). Further, the analysis showed that being single and young (ages 18–29 years) were also independently associated with poor performance on COVID-19 Knowledge. After controlling for the other independent factors, primary or secondary education also remained an independent predictor of a lower total knowledge score (Table 3).

**TABLE 3:** Multiple linear regression: Predictors of total score knowledge.

	B	SE	t	*P*-value	95.0% CI for B	
(Constant)	10.714	0.354	30.257	0.000	10.019	11.410
Outpatient	−0.507	0.161	−3.150	0.002	−0.823	−0.191
Inpatient	−1.132	0.222	−5.088	0.000	−1.569	−0.695
Gender (males vs. females)	−0.143	0.148	−0.968	0.334	−0.434	0.147
Marital status = Others	−0.092	0.315	−0.292	0.771	−0.711	0.527
Marital status = Single	−0.408	0.170	−2.399	0.017	−0.742	−0.074
Age: 18–29 (vs. 50–65+)	−0.484	0.237	−2.040	0.042	−0.949	−0.018
Age: 30–49 (vs. 50–65+)	−0.272	0.170	−1.602	0.110	−0.605	0.061
Masters or PhD	0.368	0.183	2.014	0.044	0.009	0.728
Primary school	−0.793	0.274	−2.897	0.004	−1.331	−0.255
Secondary school	−0.378	0.171	−2.205	0.028	−0.714	−0.041
Retired	−0.312	0.335	−0.931	0.352	−0.970	0.346
Student	−0.107	0.337	−0.318	0.750	−0.768	0.554
Not employed	0.253	0.173	1.460	0.145	−0.087	0.593
Place of current residence (Doha vs. outside Doha)	0.075	0.164	0.457	0.648	−0.248	0.398

B, unstandardized coefficient; SE, standard error; CI, confidence interval.

### Predictors of COVID-19 attitude: Multiple logistic regression

The regression model used the dichotomized answers to survey question 13 (Agree vs. Not) as the outcome variable and the group factor with the sociodemographic variables as the predictors; the Nagelkerke R2 showed that the model explained only 6.1% of the variance predicted by these independent factors. This best-fit model was ascertained by the Hosmer and Lemeshow test (χ^2^ = 12.01, df = 8, *p* = 0.15). The whole model gave an overall 74.9% correct rate of the outcome (Agree vs. Not). The only predictor that remained significant after controlling for the other ones was the group where the outpatient group showed 2.22 more odds of showing a positive attitude toward controlling the COVID pandemic than the general public group (Table 4).

**TABLE 4:** Multiple logistic regression: Predictors of positive attitude.

	B	SE	Wald	df	*P*-value	OR	95% CI	
							
							Upper	Lower
**Positive attitude on control of COVID-19:**								
Outpatient vs. public	0.798	0.248	10.381	1	0.001	2.222	1.367	3.611
Inpatient vs. public	−0.077	0.249	0.096	1	0.757	0.926	0.569	1.507
**Positive attitude on trust in Qatar to control the pandemic:**								
Outpatient vs. public	1.777	0.328	12.858	1	0.000	3.245	1.705	6.176
Inpatient vs. public	0.624	0.336	3.459	1	0.063	1.867	0.967	3.603

B, unstandardized coefficient; SE, standard error; OR, odds ratio; CI, confidence interval.

We obtained the same results when using the survey item 14 on the attitude regarding the trust in Qatar to control the pandemic. This best-fit model was confirmed by the Hosmer and Lemeshow test (χ^2^ = 5.15, df = 8, *p* = 0.74). The Nagelkerke R2 showed that the model explained only 7.9% of the variance predicted by these independent factors. The whole model gave an overall 84.6% correct rate of the outcome (Yes vs. No). The only predictor that remained significant after controlling for the other ones was the group where the outpatient group showed 3.25 more odds of showing a positive attitude toward control of COVID-19 pandemic than the general public group (Table 4).

### Predictors of COVID-19 practice: Multiple logistic regression

The multiple logistic regression models assessing the outcome on the practice of avoiding crowds during the pandemic (item 15, going to crowded places) showed that both patient groups were at increased odds of answering Yes compared to the general public after controlling for all factors (Table 5). Middle age (30–49 years compared to being above 50) was associated with answering No to this question. “Being employed” (compared to not employed) was also associated with a Yes answer (Table 5). This best-fit model was confirmed by the Hosmer and Lemeshow test (χ^2^ = 7.92, df = 8, *p* = 0.44). The Nagelkerke R2 showed that the model explained only 7.1% of the variance predicted by all the independent factors. The whole model gave an overall 77.5% rate of the outcome (answering Yes to question 15).

**TABLE 5:** Multiple logistic regression: Predictors of positive practice.

	B	SE	Wald	df	*P*-value	OR	95% CI	
							
							Upper	Lower
**COVID-19 practice: Going to crowded places (correct answer No)**								
Outpatient vs. public	0.706	0.233	9.187	1	0.002	2.025	1.283	3.197
Inpatient vs. public	0.663	0.280	5.603	1	0.018	1.941	1.121	3.363
Age: 18–29 vs. 50–65+	−0.273	0.260	1.100	1	0.294	0.761	0.457	1.268
Age: 30–49 vs. 50–65+	−0.902	0.347	6.745	1	0.009	0.406	0.205	0.802
Employed vs. not employed	0.604	0.256	5.584	1	0.018	1.830	1.109	3.021
Retired vs. not employed	0.528	0.534	0.979	1	0.322	1.696	0.596	4.829
Student vs. not employed	−0.040	0.552	0.005	1	0.943	0.961	0.326	2.835
**COVID-19 practice: Wearing a mask outside home (correct answer Yes)**								
Outpatient vs. public	−0.667	0.562	1.412	1	0.235	0.513	0.171	1.543
Inpatient vs. public	−1.950	0.575	11.503	1	0.001	0.142	0.046	0.439
Age: 18–29 vs. 50–65+	0.860	0.452	3.617	1	0.057	2.362	0.974	5.730
Age: 30–49 vs. 50–65+	−0.439	0.665	0.436	1	0.509	0.645	0.175	2.374
College vs. college	−1.587	1.064	2.223	1	0.136	0.205	0.025	1.648
Primary vs. college	−2.397	1.149	4.351	1	0.037	0.091	0.010	0.865
Secondary vs. college	−1.464	1.089	1.805	1	0.179	0.231	0.027	1.957

B, unstandardized coefficient; SE, standard error; OR, odds ratio; CI, confidence interval.

The regression analysis with the outcome on answer 16 (wearing masks in crowds) showed that being in the inpatient group is an independent predictor of answering No after controlling all variables. The only other factor that remained significant was having a primary education level (compared to college), which was also significantly associated with not wearing a mask in crowds (Table 5). This best-fit model was confirmed by the Hosmer and Lemeshow test (χ^2^ = 4.32, df = 8, *p* = 0.83). The Nagelkerke R2 showed that the model explained only 21.5% of the variance predicted by all the independent factors. The model gave an overall 95.1% correct rate of the outcome (correctly answering Yes to question 16).

## Discussion

This paper explores the Knowledge, Attitude, and safe practices related to COVID-19 in Qatar, comparing three groups: the general public, people with mental illness admitted to the psychiatry hospital, and mental health outpatients. This is the first paper that addresses KAP among all these groups.

The significantly higher number of male respondents from the inpatient group reflects the population of Qatar, where the male to female ratio is around 3:1. In September 2020, Qatar’s population was estimated to be 2 723 624 million, of which 1 969 032 million were males (∼72%) while 754 592 thousand were females (∼28%) (21). Male expatriates are more than threefold the female ones, while Qataris have no significant difference in sex ratio (22). Other demographics, such as age, marital status, education, and employment, can all be explained by the sociodemographic norms in Qatar. A significant majority of the population comprises male laborers in the construction industry on short-term contracts to help build the country’s infrastructure. They are often younger than 30 years of age, single, or live in Qatar without their spouses, and have lower educational attainments. Previous studies are in concurrence with the demographic findings of this study (23–26). Furthermore, mental health disorders were also found to significantly impact academic achievement and successful completion of schooling (27–30). Emerging evidence supports the correlation between mental health disorders and higher unemployment rates, which parallels the findings of unemployment rates among our patient groups compared with participants from the general public (31, 32).

### Knowledge

This study demonstrated that patient groups had inadequate Knowledge about COVID-19 compared to the public group. These findings indicate that knowledge level decreased as the acuity of mental illness increased, as those admitted to inpatient service who tend to have more acute illness had the lowest Knowledge score among the three groups. Recent studies on patients with severe mental illness (SMI) revealed poor Knowledge on COVID-19, supporting the study findings (10, 18). Among the 12 questions assessing the Knowledge of COVID-19, all three groups showed good knowledge with no significant difference regarding the two questions: using general medical masks to prevent the infection and the spread of the virus *via* respiratory droplets. However, significant differences in Knowledge were apparent among these three groups regarding the remaining ten questions. Most participants were more knowledgeable in answering those two questions due to how information was delivered regarding COVID-19 and the importance given to certain precautionary measures compared to other items in the questionnaire. Another factor that could have impacted all participants’ Knowledge might be the law mandating masks in all public places. On the other hand, other studies suggested that sedentary life and the stress of watching COVID-19 news during the pandemic might worsen the anxiety and mood symptoms in patients with mental illness and thus indirectly affect their Knowledge responses (33, 34).

Those who are single, young, and patients with lower educational levels had low COVID-19 Knowledge. These results confirm previous findings which demonstrated that people with higher educational levels showed an increase in COVID-19 awareness (2, 35, 36). Knowledge of the general public about COVID-19 symptoms, mode of transmission, and safety measures were generally very high. In China’s Hubei province, the rate of correct answers to the 12 Knowledge questions in the COVID-19 questionnaire among the general public was 90%. Their results showed significantly lower Knowledge scores among males, younger, single, lower education, and the unemployed (2). In comparison, earlier in the pandemic, a publication from China showed a considerable number of their population was not familiar with the common symptoms (37).

Findings published from Saudi Arabia, a neighboring Arabian Gulf country, were similar to the results of this study. A cross-sectional survey on Knowledge and Attitudes toward COVID-19 among their general public also showed that men and the younger people were less knowledgeable about the infection, calling for a more targeted health education program. About 44% of their population had little Knowledge about when and where to wear a mask (38). However, in the Qatari general population, 85.5% were aware that wearing a medical mask can help prevent the spread of the infection. Interestingly, this awareness was higher among patients with mental illness (88.5% of outpatients and 94% of inpatients). This might be explained by the restrictions introduced in hospitals, whereby clinic visits were stopped in favor of tele-mental health and the limitation of visits in the inpatient settings; patients were informed of these policy changes and their rationale. In another regional study in Jordan, 60.9% were considered to have adequate Knowledge, and 88.7% believed that following protective advice from health authorities effectively prevents infection (39).

### Attitude

Regarding optimism about the pandemic’s future, the outpatient group was significantly more positive than the other two groups that COVID-19 will be controlled, with 84.8% of them agreeing to the statement compared to the general public or the inpatient group. The regular contact of the outpatient with the healthcare team may have contributed to their optimism. Similarly, a significantly higher number of outpatients had confidence that Qatar would win the battle against the virus. Overall, patients with mental illness had a more positive attitude and confidence in the outcome than the general public. In the Saudi study, 94% of the general public were optimistic that the pandemic would be controlled, and 97% were confident that their government would control it (38). However, more than 50% of Jordanians did not trust the information given by their Ministry of Health or its ability to control the pandemic (39).

### Practice

The better attitude among patients with mental illness was not reciprocated with safer practice. Their adherence to avoiding crowded places was significantly lower than the general public, though still with higher adherence than not. The vast majority of all three sample populations wore a mask when leaving the house. Such a level of compliance may be attributed to the legal requirement to wear masks in public places and the fines imposed on non-adherence. The expectation is that better Knowledge results in better practices. However, applying such practices is not easy for many (40). In a Malaysian sample, those with higher Knowledge did not wear face masks or avoid crowds (3). In the Jordanian study, the majority did not wear face masks and, in fact, those with higher levels of education had lesser safe practices (39).

### Strengths and limitations

The main strength of the study is in the targeted group of participants. This is the first study to assess KAP toward COVID-19 among patients with mental illness. The study had a large multidisciplinary team that facilitated completing it within the time approved by IRB. Recruitment *via* phone was advantageous since most of those contacted had agreed to participate because they were anonymous.

Several limitations might limit the generalizability of the results. First, the sample has unequal distribution in gender. This is mainly due to the representation of the male to female ratio among the Qatar population (male to female ratio is around 3:1). Second, only the English and Arabic versions of the questionnaire were used; thus, it is not possible to generalize the results to the entire population of Qatar. Third, the majority of participants are from the labor sector who have comparatively lower levels of education. The latter might confound the responses on the KAP questionnaire. Fourth, although the study had enough power in the sample size, there is the possibility that prior knowledge about COVID-19, using text messages, and online surveys, might have affected the decision of participants to accept enrolments. Fifth, our research targeted all patients who met the inclusion criteria; we did not further assess the impact of diagnosis or symptom severity on KAP. Finally, this is a cross-sectional study covering a limited period of time, limiting the inferential conclusions about the impact of the sociodemographic variables on being in the mental health group. As the COVID-19 situation changes rapidly, changes in KAP are expected.

## Conclusion

This study demonstrated that patient groups had poorer Knowledge yet more positive Attitudes and confidence regarding the outcome of COVID-19 than the public group. Also, it was noted that Knowledge levels decreased as the acuity of mental illness increased. In addition, a more positive attitude among patient groups was evident but was not countered with greater safe practices. However, a majority of the three groups still adhered to some protective measures, such as wearing face masks. This highlights the need for targeted approaches to the awareness efforts ensuring vulnerable groups such as patients with acute mental illness receive awareness activities tailored to their needs and understandings. Rather than the currently used broader awareness targeted the whole population, there is a need for information that is specific to different age groups, and language and cognitive abilities. Non-verbal education such as using picture presentations and videos may contribute to better understanding. Engaging the family or care giver can encourage PWMI to better adhere to preventive practices. Mental healthcare professionals may incorporate KAP education in their communication with PWMI. Such interventions are likely to enhance knowledge and ensure this is reflected in better attitudes and safer practices.

## Data availability statement

The raw data supporting the conclusions of this article will be made available by the authors, without undue reservation.

## Ethics statement

The studies involving human participants were reviewed and approved by IRB at Hamad Medical Corporation. The patients/participants provided their written informed consent to participate in this study.

## Author contributions

SG submitted the research proposal and obtained the approvals. SG and IM designed the study and supervised the enrolment and procedures. YE, OA, FA, MK, and MA were involved in the recruitment and collection of data. HA-A conducted the statistical analysis. SG, IM, and HA-A wrote the manuscript. All authors reviewed the manuscript and approved the submitted version.

## Abbreviations

KAP: knowledge, attitude, and practice
HMC: Hamad Medical Corporation.
